# Compulsive Internet Use Scale: Psychometric Properties and Associations With Sleeping Patterns, Mental Health, and Well-Being in Lithuanian Medical Students During the Coronavirus Disease 2019 Pandemic

**DOI:** 10.3389/fpsyg.2021.685137

**Published:** 2021-08-26

**Authors:** Egle Milasauskiene, Julius Burkauskas, Aurelija Podlipskyte, Orsolya Király, Zsolt Demetrovics, Laurynas Ambrasas, Vesta Steibliene

**Affiliations:** ^1^Clinic of Psychiatry, Lithuanian University of Health Sciences, Kaunas, Lithuania; ^2^Laboratory of Behavioral Medicine, Neuroscience Institute, Lithuanian University of Health Sciences, Palanga, Lithuania; ^3^Institute of Psychology, ELTE Eötvös Loránd University, Budapest, Hungary; ^4^Centre of Excellence in Responsible Gaming, University of Gibraltar, Gibraltar, Gibraltar

**Keywords:** problematic internet use, validation, psychometrics, sleep, anxiety, depression, well-being, COVID-19

## Abstract

**Background:** The increase in problematic Internet use (PIU) among medical students and resident doctors during the coronavirus disease 2019 (COVID-19) pandemic may be leading to significant impairments in everyday functioning, including sleeping patterns, anxiety, depressive symptoms, and overall well-being. The Compulsive Internet Use Scale (CIUS) has been developed to assess the severity of PIU, however, it has not been elucidated whether this scale is also applicable to medical students and resident doctors. The first aim of this study was to explore the psychometric properties of the Lithuanian version of the CIUS. The second aim was to examine associations between subjectively reported mental health symptoms and PIU during the COVID-19 pandemic.

**Methods:** A total of 524 medical students and resident doctors (78.60% women, mean age 24 [SD 3] years old) participated in an online survey between December 2020 and February 2021. Participants completed the CIUS, the Pittsburgh Sleep Quality Index (PSQI) questionnaire, the Patient Health Questionnaire-9 (PHQ-9), the Generalized Anxiety Disorder Assessment-7 (GAD-7), and the WHO—Five Well-Being Index questionnaire (WHO-5).

**Results:** The confirmatory factor analysis (CFA) suggested brief versions (CIUS-5, CIUS-7, and CIUS-9) rather than the original (CIUS-14) version of the CIUS questionnaire as reliable and structurally stable instruments that can be used to measure compulsive Internet use severity in the sample of medical students and resident doctors. The most prevalent online behaviors were social media use (90.1%), online shopping (15.6%), and online gaming/gambling (11.3%). Students with higher CIUS scores reported significantly lower academic achievements during the 6 months (*r* = 0.12–0.13; *p* < 0.006), as well as more severe depressive and anxiety symptoms, worsened sleep quality, and lower sense of well-being (*r* = 0.21–0.41; *p*'s < 0.001). Both, during workdays (*d* = 0.87) and weekend (*d* = 0.33), students spent more time online than resident doctors (*p*'s < 0.001).

**Conclusion:** The brief, 5-, 7-, and 9-item versions of the Lithuanian CIUS are reliable and valid self-report screening instruments for evaluating the severity of PIU symptoms among the medical student population. Symptoms of PIU during the COVID-19 period were associated with worsened self-reported mental health and everyday functioning.

## Introduction

During the last few decades, Internet use has not only grown but has also transformed from a tool to collect and share information to a way of connecting with others and the world around us. In January 2021, 4.66 billion people were active Internet users, which means that more than 59% of the global population is currently connected to the Internet (Statista, 2021). Individuals aged 18–24 years old account for 18% of global Internet users and those aged from 25 to 34 account for 32% of global Internet users (Statista, 2021). According to a 2019 report from the Lithuanian National Department of Statistics, the daily usage of Internet among those aged 16–24 years old was 98% and those aged 25–34 years old was 95% (Lithuanian National Department of Statistics, 2019).

The general rise in Internet usage also poses a risk of online activities becoming excessive such as gaming/gambling, streaming, watching pornography, and shopping (Fineberg et al., 2018). Problematic Internet use (PIU) is defined as the excessive and compulsive use of online activities and services which have addictive potential associated with marked functional impairment (Király et al., 2015; Ioannidis et al., 2016, 2019).

Following the spatial distancing recommendations of World Health Organization (2020) imposed by many governments around the world in response to the coronavirus disease 2019 (COVID-19) pandemic, many international experts have expressed concern over worsening PIU. To address this, a group of international experts has recommended guidelines for diminishing the risks of increased Internet usage, including encouraging individuals to reach out to mental health professionals if they experienced high levels of distress or significant difficulties controlling their Internet use or specific online activities (Király et al., 2020). Risk factors contributing to PIU include well-known risk factors such as age, sex, and mental health problems, as well as new risk factors brought about by the COVID-19 pandemic such as higher distress or diminished coping. As reported in a recent study by Sun et al. (2020), coping behaviors, such as Internet use, alcohol consumption, and smoking, during the COVID-19 pandemic increased the risk for subsequent substance use disorders and Internet addiction (Sun et al., 2020). In recent years, especially during the COVID-19 pandemic, medical students and resident doctors have experienced an unprecedented expansion of Internet use for online learning, work, and communication (Almomani et al., 2021). The development of electronic patient records, online networking, and training of healthcare professionals has provided advantages for information sharing, learning, and decision-making (Gill et al., 2013). Medical students and doctor residents are among the more vulnerable population because they have to study and work long day and night hours, in most cases without a structured time schedule. At the same time, they have high inspiration for professional development and unlimited access to use Internet-based technologies. However, despite numerous advantages of Internet-based technologies in medical training and the healthcare system, there is still a growing body of evidence that excessive Internet use could lead to PIU among healthcare professionals and cause comorbid problems related to mental health. The prevalence rate for PIU was reported at 7.2% in the general population (Pan et al., 2020) compared with 9.7% among healthcare professionals (Buneviciene and Bunevicius, 2020) and 30.1% among medical students (Zhang et al., 2018). It is well-recognized that medical students and resident doctors are at a greater risk for mental health problems when compared with the general population (Puthran et al., 2016; Erschens et al., 2019; Zeng et al., 2019; Zhou et al., 2020). Moreover, the sudden shift from in-person learning to online education among the medical student population during the COVID-19 pandemic period was associated with higher odds of having generalized anxiety and being depressed (Essangri et al., 2020; Moitra et al., 2021; Nishimura et al., 2021). Studies also suggest that for resident doctors, excessive Internet use might emerge as a compensatory coping behavior for mental health problems (Ueno et al., 2020). A study examining the correlation between PIU, mental health, and sleep quality among Iranian medical students found that PIU was directly and positively connected to depression (*r* = 0.44; *p* < 0.001), anxiety (*r* = 0.45; *p* < 0.001), and stress (*r* = 0.40; *p* < 0.001) (Shadzi et al., 2020). Furthermore, in their meta-analysis of 22,778 resident doctors, Low et al. (2019) found that the aggregate prevalence of burnout was 51.0%. As a result, mental health problems often severely affect academic performance, dropout rates, and professional development (Dyrbye et al., 2006; Khan et al., 2016; Taha et al., 2019). PIU has been linked not only with sleep problems (You et al., 2021) but also with suicidal behavior mediated by sleep disturbances (Guo et al., 2018).

Reliable and validated tools would allow better identification of the problematic behavior among future medical professionals. However, in Lithuania, a scarcity of available instruments remains for investigating PIU. Most of the existing studies conducted in Lithuania focused on children and teenagers (Blinka et al., 2015; Škarupová et al., 2015; Ustinavičiene et al., 2016), and only two investigated PIU symptoms among students using a psychometrically sound nine-item PIU questionnaire, namely, the PIUQ-9 (Burkauskas et al., 2020; Gecaite-Stonciene et al., 2021). Consequentially, it is of crucial importance to update the toolbox of PIU measurements to investigate the symptomatology in specific samples of individuals who might be particularly vulnerable to the phenomena.

The Compulsive Internet Use Scale (CIUS) has been proposed as a tool to assess the severity of Internet addiction and compulsive, pathological, or problematic online behaviors. In the review conducted by Laconi et al. (2014), over 40 scales were identified as measuring PIU at the time. However, the CIUS was regarded as one of the shortest questionnaires in comparison with other scales measuring PIU severity, while having good psychometric properties at the same time. Thus, this scale might be of particular use for screening medical professionals for PIU as it takes about 3–5 min to complete it.

The CIUS was design based on the Diagnostic and Statistical Manual of Mental Disorders-IV (DSM-IV) criteria for Dependence and Pathological Gambling and also includes general features of behavioral addictions which were added based on the recommendations by Griffiths (1999) and the results of a qualitative study executed by the author of the scale (Meerkerk et al., 2003). The scale includes typical symptoms of compulsive Internet use such as the inability to control Internet use, mental and behavioral preoccupation with online activities, agitation associated with the inability to go online, mood change, and conflicts with significant others over Internet use (Meerkerk et al., 2009).

The CIUS has been adapted and psychometrically tested in various languages and specific groups of individuals showing good psychometric properties (Dhir et al., 2015; Lopez-Fernandez et al., 2019). However, there is still debate on the CIUS factor structure, with some studies proposing the CIUS as a one-dimensional instrument (Khazaal et al., 2012; Guertler et al., 2014; Lopez-Fernandez et al., 2019) and others finding better compatibility for a three-factor model consisting of CIUS—Absorption, CIUS—Priorities, and CIUS—Mood regulation (Alavi et al., 2011; Yong et al., 2017). Furthermore, a study by Lopez-Fernandez et al. (2019) found that short versions of the instrument (CIUS-5, CIUS-7, and CIUS-9) provide an even better one-factor structure than the original 14-item questionnaire. To date, the CIUS psychometric properties have not been analyzed in Lithuania among the populations of medical students and resident doctors.

Thus, the current study aimed to examine the psychometric properties of the Lithuanian version of CIUS in a sample of medical students and resident doctors. An additional aim was to examine the relationship between PIU and mental health problems, such as subjective sleep quality, depression and anxiety symptoms, and overall well-being.

## Materials and Methods

### Study Procedure

This cross-sectional study was conducted in the Lithuanian University of Health Sciences (LUHS) departments between December 2020 and February 2021 (during the COVID-19 pandemic). Using the official university mailing system and social media groups for medical students and resident doctors, study participants were invited to fill out an online survey available through Google Forms. Before starting the survey, participants had to provide online informed consent to participate in the study by ticking the appropriate answer “agree/disagree.” There were no incentives for study participants on completion.

The survey comprised of scales on compulsive Internet use CIUS, sleeping patterns (Pittsburgh Sleep Quality Index, PSQI), depression symptoms (Patient Health Questionnaire-9, PHQ-9), anxiety symptoms (Generalized Anxiety Disorder Assessment-7, GAD-7), and general well-being (WHO—Five Well-Being Index, WHO-5). The sociodemographic questionnaire was developed by the authors and included data on age, sex, living conditions, family situation, physical activity, academic achievements, participation in academic classes, and Internet use habits of participants including a question whether individuals think that their online behavior is problematic (“yes”/ “no”). Overall, the survey included 62 questions, with a completion time ranging from 18 to 25 min with possibly minimal chances for “survey fatigue.” The approval from the Bioethics Committee of the LUHS (No. BEC-LSMU [R]-18) was received and the study was executed in accordance with the principles of the Declaration of Helsinki.

### Measures

The CIUS is a self-report 14-item scale for the assessment of the severity of Internet addiction and/or compulsive, pathological, or other Internet use which could be considered as problematic. Each question utilizes a 5-point Likert scale, ranging from 0 (“never”) to 4 (“very often”) and results in a total score ranging from 0 to 56. A higher score indicates a higher severity of PIU. Shortened versions of the CIUS were proposed by Lopez-Fernandez et al. (2019) (with CIUS-9 including items 1, 3, 4, 5, 7, 9, 11, 12, 14; CIUS-7 including items 1, 3, 5, 7, 9, 11, 12; and CIUS-5 including items 1, 3, 5, 11, 12).

The author of the CIUS approved the use of the scale within the current study (Meerkerk et al., 2009). A double back-translation procedure was used to translate the CIUS questions from English to Lithuanian. An experienced psychiatrist with the knowledge of PIU terminology (VS) performed the original translation from English to Lithuanian. Bilingual (English and Lithuanian) native speakers from the registered academic translation service provider conducted the back translation from Lithuanian to English. Significant differences in the back translation as compared with the original CIUS version were discussed to reach the final consensus.

Sleep patterns of participants were rated using the PSQI, which is a self-rated questionnaire for the assessment of subjective sleep quality over 1 month (Buysse et al., 1989). The PSQI consists of 19 questions each ranging from 0 to 3 points, where 0 points indicate “no difficulties” and 3 points indicate “severe sleep problems.” Answers are summed into seven components of a global PSQI: subjective sleep quality, sleep latency, sleep duration, habitual sleep efficiency, sleep disturbances, use of sleeping medication, and daytime dysfunction. The global PSQI score is the sum of the seven components and ranges from 0 to 21. The Lithuanian version of the PSQI (Varoneckas, 2003) showed acceptable internal consistency in this study sample (Cronbach's α was 0.71).

A brief self-rated questionnaire, the PHQ-9 was used for measuring the severity of depressive symptoms (Kroenke et al., 2001). Nine items of the questionnaire are based on the depression diagnostic criteria of the DSM-IV; each of the nine items scores from 0 (“Not at all”) to 3 (“Nearly every day”). The total score equals the sum of each of the nine items and ranges from 0 to 27, where the higher scores indicate more severe depressive symptoms. Internal consistency of the scale in this study sample is considered good (Cronbach's α 0.84).

The GAD-7 was used to measure the severity of anxiety symptoms (Spitzer et al., 2006). In this seven-item questionnaire, participants have to provide their responses on a 4-point Likert scale ranging from 0 (“Not at all”) to 3 (“Nearly every day”). The total score is the sum of all seven answers and ranges from 0 to 21; the higher sum score indicates more severe anxiety symptoms. Internal consistency of the scale in our study sample is considered good (Cronbach's α was 0.91).

Current subjective psychological well-being was assessed using the WHO-5 (World Health Organization, 1998; Topp et al., 2015). This instrument consists of five statements about the period of the past 2 weeks and responses range from 0 (“At no time”) to 5 (“All of the time”). The sum score ranges from 0 to 25. Because scales measuring health-related quality of life are conventionally translated to a percentage scale from 0 (absent) to 100 (maximal), it is recommended to multiply the sum score by four to give the final index: 0 means the worst well-being and 100—the best imaginable well-being. Internal consistency of the scale is in this study sample is considered good (Cronbach's α was 0.88).

### Statistical Analysis

Descriptive statistics, internal consistency (Cronbach's α), and exploratory and confirmatory factor analyses (EFA and CFA, respectively), were employed to analyze the reliability and the structural validity of the questionnaire. EFAs were performed to analyze the factor structure of the CIUS by using a common factor method including oblique Promax rotation. Principal axis factoring involved data structure analysis that examined shared variances that are considered unique to individual measurement. Items with a factor loading of 0.3 were considered acceptable (Streiner et al., 2015). The test of sphericity suggested by Bartlett was used to determine whether the correlation between the items was adequate for conducting the factor analysis, with a *p* < 0.05 indicating suitability for structure detection (Tabachnick et al., 2007). The Kaiser–Meyer–Olkin (KMO) index was used to test the sampling adequacy (KMO index > 0.6) (Tabachnick et al., 2007). A scree plot was used for the factor pattern interpretation to decide on the number of the CIUS factors.

Later, CFA was performed for the validation of the factor structure considered in EFA using the maximum likelihood method. In this study, we planned to compare the fit indices of the one- and three-factor models and the proposed one-factor solution of the shortened versions of the instrument (i.e., CIUS-9, CIUS-7, and CIUS-5). The Analysis of Moment Structures (AMOS) 22.0 software was used to test the one-factor model of CIUS-14, the CIUS-9, the CIUS-7, and the CIUS-5, and the three-factor model of CIUS-14 by CFA.

Proposed thresholds for the CFA fit indices were: comparative fit index (CFI) > 0.90 adequate and > 0.95 good, Tucker–Lewis Index (TLI) > 0.90 adequate and > 0.95 good, normed fit index (NFI) > 0.90 adequate and > 0.95 good, root-mean-square error of approximation (RMSEA) < 0.08, χ2/df with desired range of values 2–5 (Hooper et al., 2008; Brown, 2015).

Finally, using the two-tailed Student's *t*-test and the Fisher's χ^2^ test, we compared the CIUS scores and the sociodemographic characteristics and subjective psychological assessments between students and resident doctors. Pearson's correlational analysis was used to assess associations between the CIUS scores and subjective psychological measures. Statistical analyses were performed with the Statistical Package for the Science Software v.22 (SPSS, Chicago, IL, United States). The level of significance was set at *p* < 0.05.

## Results

In total, 1,064 resident doctors and 1,650 medical students were invited to participate in the study. Of those invited to participate, significantly more responses were received from the medical student group than the resident doctors (20.8 vs. 16.9%, respectively; *p* < 0.05); and the respondents were younger when compared with non-respondents (*p* < 0.05). The response rate was not associated with the sex of participants. Overall, the response rate was 19.3% with a total of 524 individuals who completed the survey.

Of the 524 study participants, 65.6% were medical students and 34.4% were resident doctors and the overall group means age was 23.7 (3.1) years old (Table 1). The majority of the sample was women, living with a partner or family member, married or in a partnership, and non-smokers. The mean length of Internet use was 4.89 (SD = 2.61) h/day on workdays and 5.07 (SD = 2.68) h/day on weekends. The most prevalent online behavior of the participants was social media use, followed by academic uses, online shopping, and online gaming/gambling. There was an inverse correlation between PIU as measured by the CIUS and academic achievements among the whole study group (*r* = −0.345; *p* < 0.001).

**Table 1:** Sociodemographic characteristics and self-reported psychological assessments in study participants.

**Characteristics**	**All, *n* = 524**
Age, years; mean (SD)	23.7 (3.1)
**Types of studies, n (%)**
Medical student	344 (65.6)
Doctor resident	180 (34.4)
**Sex, n (%)**
Women	412 (78.6)
Men	112 (21.4)
**Living condition, n (%)**
Alone	174 (33.2)
With partner/family members	350 (66.8)
**Marital state, n (%)**
Single	266 (43.1)
Married/partnership	298 (56.9)
**Smoking, n (%)**
Yes	112 (21.4)
No	412 (78.6)
Physical activity, hours/day; mean (SD)	0.80 (0.66)
**Time using internet**
Workdays, hours/day; mean (SD)	4.89 (2.61)
Weekends, hours/day; mean (SD)	5.07 (2.68)
**The online behavior, n (%)**
Academic	406 (77.5)
Online gaming/gambling	59 (11.3)
Online shopping	82 (15.6)
Online social media	472 (90.1)
Online relationship/partnership	4 (0.8)
Online pornography	6 (1.1)
Streaming (movies/shows etc.)	19 (3.6)
**Academic achievements, n (%)**
Excellent/higher than average	228 (43.5)
Lower than average/poor	296 (56.5)
**Participation in academic classes, n (%)**
In the most of the classes	454 (86.6)
Only mandatory classes/not participate at all	70 (13.4)
Use of medications to treat depression/anxiety/cope with stress	44 (8.4)
Five Well-Being Index WHO-5, total score; mean (SD)	51.9 (18.9)
Global PSQI index, mean (SD)	6.6 (3.0)
PHQ-9, total score; mean (SD)	9.1 (5.7)
GAD-7, total score; mean (SD)	7.4 (5.1)

*SD, standard deviation; WHO-5, World health Organization—Five Well-Being Index; PSQI, Pittsburgh Sleep QualityIndex; PHQ-9, Patient Health Questionnaire-9, GAD-7, Generalized Anxiety Disorder Assessment-7*.

Significant differences between the students and resident doctors were observed in time spent online during workdays [*M* = 5.59, *SD* = 2.61 vs. *M* = 3.56, *SD* = 2.03; *t*_(522)_ = 9.79, *p* < 0.001, large effect *d* = 0.87] and weekends [*M* = 5.37, SD = 2.69 vs. *M* = 4.51, SD = 2.56; *t*_(522)_ = 3.54, *p* < 0.001, small effect *d* = 0.33]. However, differences between the aforementioned groups were not significant in the case of the CIUS total score and for other mental health indices as measured by the PHQ-9, the GAD-7, the PSQI index, and the WHO-5 (*p*'s > 0.05).

### Psychometric Properties of the Lithuanian Version of the CIUS in the Sample of Medical Students and Doctor Residents

The Lithuanian version of the 14-item CIUS had a very high internal consistency of α = 0.90 for the sample of Lithuanian medical students and resident doctors. The total correlation for all items is shown in Table 2. Internal consistency for the three-factor model was good with α ranging from 0.82 to 0.85. Internal consistency of the CIUS-9 and the CIUS-7 was also good with α of 0.84 and 0.81, respectively. Internal consistency of the CIUS-5 was considered acceptable with α of 0.73.

**Table 2:** Means, robust estimator, and intercorrelations of the compulsive internet use scale items.

**Item**	**Mean (SD)**	**Huebr's M estimator**		**1**	**2**	**3**	**4**	**5**	**6**	**7**	**8**	**9**	**10**	**11**	**12**	**13**	**14**	**15**
1. CIUS total	22.1 (10.5)	22.1		1														
2. CIUS1	2.13 (1.09)	2.17		0.753^*^	1													
3. CIUS2	2.09 (1.09)	2.15		0.762^*^	0.729^*^	1												
4. CIUS3	1.03 (1.13)	0.94		0.516^*^	0.361^*^	0.383^*^	1											
5. CIUS4	1.25 (1.10)	1.19		0.646^*^	0.453^*^	0.505^*^	0.325^*^	1										
6. CIUS5	2.06 (1.14)	2.11		0.635^*^	0.455^*^	0.467^*^	0.197^*^	0.395^*^	1									
7. CIUS6	1.09 (1.08)	1.02		0.688^*^	0.432^*^	0.442^*^	0.343^*^	0.419^*^	0.372^*^	1								
8. CIUS7	0.99 (1.09)	0.91		0.693^*^	0.435^*^	0.417^*^	0.371^*^	0.441^*^	0.366^*^	0.656^*^	1							
9. CIUS8	2.21 (1.26)	2.27		0.718^*^	0.575^*^	0.585^*^	0.397^*^	0.305^*^	0.414^*^	0.450^*^	0.419^*^	1						
10. CIUS9	1.66 (1.19)	1.71		0.725^*^	0.549^*^	0.544^*^	0.305^*^	0.349^*^	0.447^*^	0.461^*^	0.396^*^	0.722^*^	1					
11. CIUS10	0.89 (1.07)	0.99		0.693^*^	0.395^*^	0.438^*^	0.351^*^	0.438^*^	0.356^*^	0.493^*^	0.532^*^	0.385^*^	0.438^*^	1				
12. CIUS11	0.88 (1.10)	0.90		0.628^*^	0.412^*^	0.430^*^	0.281^*^	0.411^*^	0.342^*^	0.355	0.409^*^	0.296^*^	0.338^*^	0.557^*^	1			
13. CIUS12	2.49 (1.09)	2.57		0.675^*^	0.493^*^	0.479^*^	0.158^*^	0.374^*^	0.430^*^	0.311^*^	0.328^*^	0.452^*^	0.440^*^	0.352^*^	0.362^*^	1		
14. CIUS13	2.34 (1.26)	2.44		0.601^*^	0.413^*^	0.363^*^	0.121^*^	0.286^*^	0.339^*^	0.259^*^	0.297^*^	0.339^*^	0.376^*^	0.323^*^	0.323^*^	0.740^*^	1	
15. CIUS14	1.01 (1.10)	0.93		0.626^*^	0.349^*^	0.357^*^	0.249^*^	0.390^*^	0.352^*^	0.487^*^	0.459^*^	0.302^*^	0.360^*^	0.403^*^	0.403^*^	0.360^*^	0.364^*^	1

* *Correlation is significant at the 0.010 level (two-tailed). CIUS, Compulsive Internet Use Scale*.

**Figure 1:**
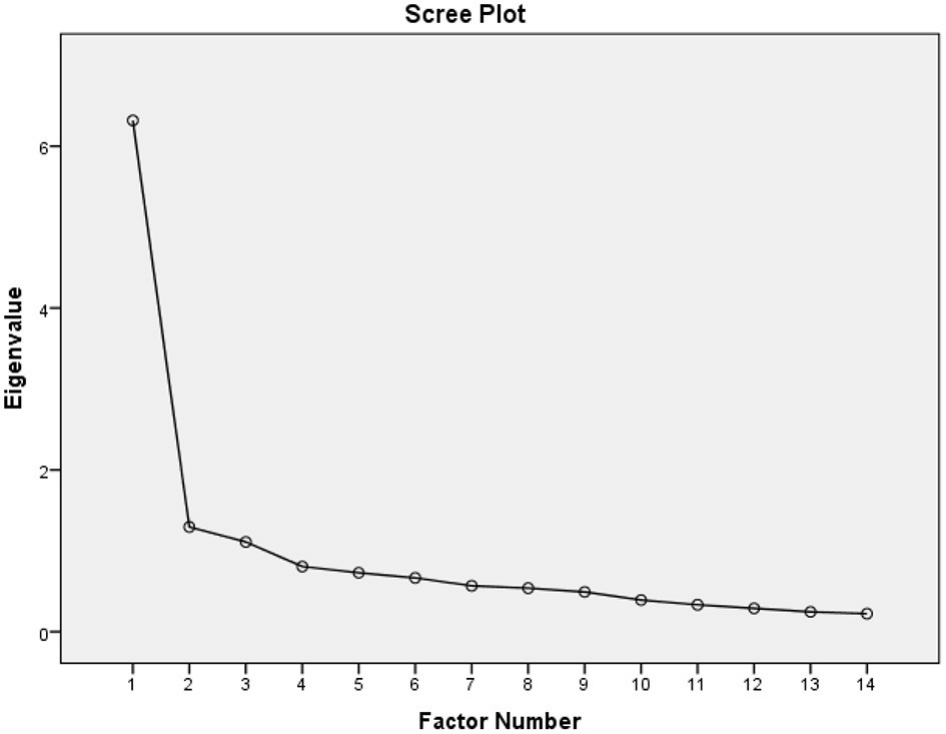
Scree plot illustrating the factor loadings with parallel analysis of the Lithuanian version of the Compulsive Internet Use Scale (CIUS).

The KMO of 0.901 indicated the applicability of data for the factor analysis. The test of sphericity (*p* < 0.001) suggested by Bartlett supported the factorability of the correlation matrix. EFAs were performed including the solutions of one- and three-factor models (Table 3). The factor loadings for the one-factor model ranged from 0.460 to 0.757 and factor loadings for the three-factor model ranged from 0.344 to 0.940. A one-factor model was determined to explain 45.1% and the three-factor model was determined to explain 62.3% of the total variance, which is considered sufficient for a coherent construct of the CIUS. While the scree plot (Figure 1) indicated a one-factor structure, results of the EFA suggested both models as acceptable.

**Table 3:** Factors loadings of each item of the CIUS.

**Compulsive internet use scale**	**One-factor model**	**Three-factor model**		
	**F1**	**F1**	**F2**	**F3**
1. Do you find it difficult to stop using the Internet when you are online?	0.745		0.638	
2. Do you continue to use the Internet despite your intention to stop?	0.757		0.645	
3. Do others (e.g., partner, children, parents) say you should use the Internet less?	0.406		0.344	
4. Do you prefer to use the Internet instead of spending time with others (e.g., partner, children, parents)?	0.611	0.513		
5. Are you short of sleep because of the Internet?	0.596	-	-	-
6. Do you think about the Internet, even when not online?	0.663	0.682		
7. Do you look forward to your next Internet session?	0.666	0.770		
8. Do you think you should use the Internet less often?	0.692		0.940	
9. Have you unsuccessfully tried to spend less time on the Internet?	0.702		0.732	
10. Do you rush through your (home) work in order to go on the Internet?	0.664	0.737		
11. Do you neglect your daily obligations (work, school, or family life) because you prefer to go on the Internet?	0.588	0.577		
12. Do you go on the Internet when you are feeling down?	0.635			0.860
13. Do you use the Internet to escape from your sorrows or get relief from negative feelings?	0.545			0.829
14. Do you feel restless, frustrated, or irritated when you cannot use the Internet?	0.583	0.639		
Eigen value	6.32	6.32	1.30	1.11
% of variance	45.1	45.1	9.25	7.92
Cumulative % of variance	45.1	45.1	54.4	62.3

Thus, CFAs were then used to confirm the construct dimensionality of the CIUS by testing the solutions of both one-factor and three-factor models. The distribution of the CIUS scores was normal (absolute values skewness from 0.043 to 0.613 and kurtosis from 0.418 to 1.257) (Kim, 2013) and the maximum likelihood estimation method was applied for the CFAs. Both the one-factor model (RMSEA = 0.140, RMSEA 90% CI = 0.131–0.148; CFI = 0.774; NFI = 0.758; TLI = 0.733; χ^2^/*df* = 11.2) and the three-factor model (RMSEA = 0.094, RMSEA 90% CI = 0.085–0.104; CFI = 0.911; NFI = 0.895; TLI = 0.889; χ^2^/*df* = 5.67) showed unacceptable fit. However, we also tested the one-factor solution of the shortened versions of the CIUS as proposed by Lopez-Fernandez et al. (2019). All three versions of the shortened CIUS, namely, the CIUS-9, the CIUS-7, and the CIUS-5 showed acceptable model fit for the one-factor solutions according to the CFAs (Table 4).

**Table 4:** Confirmatory Factor Analyses (CFA) were conducted using the original CIUS.

	**α**	**χ^**2**^/df**	**df**	**χ^**2**^**	***p***	**CFI**	**TLI**	**NFI**	**RMSEA**	**RMSA 90%CI**	**p_**close**_**
F1—CIUS-14	0.904	11.21	77	862,761	<0.001	0.774	0.733	0.758	0.140	0.131–0.148	<0.001
F3—CIUS-14	0.837–0.845	5.67	62	351.237	<0.001	0.911	0.889	0.895	0.094	0.085–0.104	<0.001
F1—CIUS-9	0.844	3.65	27	98.513	<0.001	0.948	0.930	0.930	0.071	0.056–0.087	0.010
F1—CIUS-7	0.807	3.73	14	52.153	<0.001	0.960	0.940	0.947	0.072	0.052–0.094	0.037
F1—CIUS-5	0.727	4.58	5	22.882	<0.001	0.965	0.930	0.956	0.083	0.050–0.118	0.049

*F1, one-factor model. F3, a three-factor model (Item 5 is not included due to insufficient factor loading on EFA). CIUS, the Compulsive Internet Use Scale (number represents the sum of items). χ^2^, Chi-square; df, degrees of freedom; CFI, comparative fit index; TLI, Tucker–Levis Index; RMSEA, root-mean-square error of approximation; 90% CI, 90% confidence interval of the RMSEA; p_close_, provides a one-sided test of the null hypothesis that the RMSEA is equal to 0.05 in the population*.

### PIU in Relation to Sleeping Quality, Mental Health, and Well-Being in Lithuanian Medical Students and Resident Doctors

Analysis of the associations among self-reported psychological measures and PIU showed that higher CIUS total scores were associated with more severe depressive and anxiety symptoms, worsened sleep quality, and lower overall well-being (Table 5). Answers (“yes”/ “no”) to the question on whether participants considered their online behavior as problematic also positively correlated with the CIUS total score (Table 5).

**Table 5:** Pearson's correlations between the CIUS scores and depression, anxiety, subjective sleep, and well-being indices.

	**CIUS-9**	**CIUS-7**	**CIUS-5**
	**r (*p*)**	**r (*p*)**	**r (*p*)**
*Depression symptoms as measured with the Patient Health Questionnaire-9*	0.410 (<0.001)	0.411 (<0.001)	0.410 (<0.001)
*Anxiety symptoms as measured with the Generalized Anxiety Disorder Scale 7*	0.223 (<0.001)	0.216 (<0.001)	0.213 (<0.001)
*Question on whether the participants considered their online behavior as problematic (“yes”/* “*no”)*	0.470 (<0.001)	0.467 (<0.001)	0.443 (<0.001)
**Subjective sleep quality**			
Subjective sleep quality	0.242 (<0.001)	0.251 (<0.001)	0.254 (<0.001)
Sleep latency	0.118 (0.007)	0.127 (0.003)	0.125 (0.004)
Sleep duration	0.100 (0.022)	0.095 (0.029)	0.113 (0.010)
Habitual sleep efficiency	0.025 (0.566)	0.052 (0.238)	0.057 (0.195)
Sleep disturbances	0.174 (<0.001)	0.163 (<0.001)	0.159 (<0.001)
Use of sleeping medication	0.146 (0.001)	0.140 (0.001)	0.151 (0.001)
Daytime dysfunction	0.334 (<0.001)	0.333 (<0.001)	0.337 (<0.001)
Global Pittsburg Sleep Quality Index, total score	0.271 (<0.001)	0.279 (<0.001)	0.287 (<0.001)
**Subjective psychological well-being**			
I have felt cheerful in good spirits	−0.237 (<0.001)	−0.233 (<0.001)	−0.235 (<0.001)
I have felt calm and relaxed	−0.174 (<0.001)	−0.188 (<0.001)	−0.181 (<0.001)
I have felt active and vigorous	−0.267 (<0.001)	−0.265 (<0.001)	−0.261 (<0.001)
I woke up feeling fresh and rested	−0.207 (<0.001)	−0.219 (<0.001)	−0.233 (<0.001)
My daily life has been filled with things that interest me	−0.234 (<0.001)	−0.243 (<0.001)	−0.246 (<0.001)
Well-Being Index (WHO-5)	−0.271 (<0.001)	−0.279 (<0.001)	−0.281 (<0.001)

## Discussion

The main finding of the current study was the confirmation of the use and good psychometric characteristics of the shortened CIUS scales in a population of medical students and resident doctors. Symptoms of PIU were associated with worsened anxiety and depression symptoms, sleep patterns and lowered self-reported sense of well-being.

Results of the internal consistency of the Lithuanian CIUS versions are in line with other studies which found the CIUS-9, CIUS-7, and CIUS-5 internal consistency scores ranging between 0.74 and 0.99 (Lopez-Fernandez et al., 2019). Good psychometric properties of the instrument allow it to be used by health professionals to identify and monitor PIU symptom severity in medical students and resident doctors. Having a psychometrically valid instrument is of crucial importance for the accurate assessment of PIU within vulnerable and at-risk populations. The one-factor model solution for CIUS-9, CIUS-7, and CIUS-5 through CFA complements the analysis performed by Lopez-Fernandez et al. (2019) who also found CIUS-14 in CFA to provide a poor global overall model fit. However, there was confirmatory evidence of the convergent construct validity of CIUS, based on the associations of the total score of questionnaires with the mental health, sleep patterns, and well-being indices.

The majority of the study participants reported an academic purpose for Internet use, however, PIU was inversely associated with academic achievements in this sample. Internet use for entertainment purposes was found to be a significant predictor of Internet addiction among women non-medical students (Abdel-Salam et al., 2019). Ueno et al. (2020) also suggested that immersion into virtual reality could be used as a coping mechanism for large academic load and negative psychological states (Ueno et al., 2020).

In contrast, in medical students and resident doctors, PIU severity was associated with several mental health problems, including worse sleep quality, lower subjective feeling of psychological well-being, higher rates of depression, and anxiety compared with medical students and resident doctors without PIU. The effect size of these results varied from small in the case of anxiety, sleep quality, and well-being indices to medium in the case of depression symptoms. A recent study conducted in Lithuania in the general student population showed significant associations between PIU (as measured by the PIUQ-9 questionnaire), anxiety, and depressive symptoms (Gecaite-Stonciene et al., 2021). Similarly, symptoms of anxiety, depression, and sleep disturbances were associated with PIU among the medical population in studies conducted in other countries (Capetillo-Ventura and Juárez-Treviño, 2015; Younes et al., 2016; Grover et al., 2019; Shadzi et al., 2020). Recent studies found that PIU correlates with sleep problems, depression, and anxiety in non-medical populations as well (de Vries and Nakamae, 2018; Khazaal et al., 2021).

The study results suggest a relationship between a higher CIUS total score and lower subjective sleep quality. Kalmbach et al. (2017) suggest that sleep deprivation among physicians-in-training leads to higher medical error rates and may also have a negative effect on the mental health of doctors (Gecaite-Stonciene et al., 2021).

We did not find any significant associations between sex, age, living conditions, marital status, time spent using the Internet, smoking, physical activity, and use of psychotropic medications and PIU, whereas other studies found significant positive correlations between Internet addiction and urban living (Cao et al., 2011; Stavropoulos et al., 2013; Yasuma et al., 2019), and gaming on the Internet (Tsumura and Kanda, 2018) and time spent online (Laconi et al., 2019) among non-medical populations.

While time spent online is an important contributor to PIU, several studies (Mazhari, 2012; Orben and Przybylski, 2019; Coyne et al., 2020), including the one by the CIUS author (Meerkerk et al., 2009), suggest that specific activities online (e.g., gaming or watching pornography) might contribute more to the PIU symptomatology than the actual time spent online. The most prevalent activity online in this study sample was academic search and social media use. Other studies also suggest that time spent on these activities is not directly related to PIU (Mazhari, 2012; Orben and Przybylski, 2019; Coyne et al., 2020). However, this study reveals the negative impact of PIU on both mental health and sleep quality, which is in line with the results of Shadzi et al. (2020).

However, significant differences between the student and resident doctor groups emerged in time spent online. During workdays, students tend to spend ~2 h more time online than resident doctors. The difference persists during weekends, although the time difference is much lower (~45 min). This difference could be explained by the roles and responsibilities of the groups, as resident doctors are usually full or part-time workers and have to take care of other duties which are not online both during workdays and weekends.

Even though we were the first to validate a Lithuanian language version of the short CIUS scales and use it in assessing associations with mental health problems among the students of medical faculty and resident doctors in Lithuania, the study should be interpreted in the context of its design and limitations.

The results depended on self-reported data and cannot be generalized as only a small sample of medical students and resident doctors from Lithuania were included. Also, this was a cross-sectional study and causality could not be established. Data collection may have been affected by selection bias, as questionnaires were distributed only through online platforms, so participants who use the Internet more often were possibly more likely to fill out the online questionnaire and were also more likely to be affected by PIU. In this survey, we had not been collecting the COVID-19-related medical status of participants. Living with or treating a person who was (or is) tested positive with COVID-19 or experiencing a loss because of the COVID-19 may have been important stressors (to control) affecting the mental health of participants during COVID-19 and probably the Internet use practices of participants.

We have chosen the CIUS for its brevity and the possibility to capture key symptoms of PIU such as loss of control, preoccupation with online activities, and mood change. As measured with the CIUS, PIU was found to be associated with higher levels of anxiety, depression, sleep problems, lower subjective feeling of psychological well-being, and poorer academic performance. The strongest effect was observed for depression symptoms.

It remains unknown whether PIU predicts subsequent psychiatric disorders or if the causality is reversed. The findings of this study should also be taken into account by the university hospital administrative staff. Despite the numerous advantages of Internet use, if used in a problematic way, Internet use could be associated with poor labor productivity, a higher prevalence of medical errors, and a risk to the safety of patients.

In conclusion, the Lithuanian versions of the CIUS-9, the CIUS-7, and the CIUS-5 are reliable and valid instruments for evaluating the severity of PIU symptoms among the medical student population. In medical students and resident doctors, studying during the COVID-19 pandemic, symptoms of PIU were associated with worsened subjectively evaluated mental health and everyday functioning.

## Data Availability Statement

The raw data supporting the conclusions of this article will be made available by the authors, without undue reservation.

## Ethics Statement

The studies involving human participants were reviewed and approved by Bioethics Committee of Lithuanian university of Health sciences. The participants provided their written informed consent to participate in this study.

## Author Contributions

JB and VS designed the study. EM and LA collected and analyzed the data. AP: statistical analyses. EM, JB, LA, AP, ZD, OK, and VS: drafted and edited the manuscript. All authors contributed to the manuscript and approved the final version.

## Conflict of Interest

For the past several years, JB has been serving as a consultant to Cogstate, Ltd. VS reported being a consultant to SignantHealth and received personal fees from Lundbeck, Sanofi, Servier, Johnson & Johnson, KRKA, and grants from the Research Council of Lithuania. The remaining authors declare no commercial or financial relationships could be construed as a potential conflict of interest.

## Publisher's Note

All claims expressed in this article are solely those of the authors and do not necessarily represent those of their affiliated organizations, or those of the publisher, the editors and the reviewers. Any product that may be evaluated in this article, or claim that may be made by its manufacturer, is not guaranteed or endorsed by the publisher.
